# Combined poor diabetes control indicators are associated with higher risks of diabetic retinopathy and macular edema than poor glycemic control alone

**DOI:** 10.1371/journal.pone.0180252

**Published:** 2017-06-29

**Authors:** Eva K. Fenwick, Jing Xie, Ryan E. K. Man, Charumathi Sabanayagam, Lyndell Lim, Gwyn Rees, Tien Y. Wong, Ecosse L. Lamoureux

**Affiliations:** 1 Centre for Eye Research Australia, Royal Victorian Eye and Ear Hospital, University of Melbourne, Melbourne, Australia; 2 Singapore Eye Research Institute, Singapore National Eye Centre, Singapore, Singapore; 3 Duke NUS Medical School, National University of Singapore, Singapore, Singapore; Boston University School of Medicine, UNITED STATES

## Abstract

**Purpose:**

To examine the association of individual and combined indicators of diabetes control with diabetic retinopathy and diabetic macular edema.

**Materials and methods:**

In this clinical, cross-sectional study, 613 adults with type 2 diabetes (372 any diabetic retinopathy; 183 any diabetic macular edema) were examined. Diabetic retinopathy was assessed from fundus photographs; diabetic macular edema from Ocular Coherence Tomography scans; and HbA_1c_ and serum lipid values from fasting blood samples. Poor glucose control was defined as Hb_A1c_≥7%; poor blood pressure control as SBP≥130/DBP≥80; and poor lipid control as total cholesterol:HDL ratio≥4.0. The association of poor glucose control, poor blood pressure control and poor lipid control alone and in combination (poor glucose & blood pressure control; poor glucose & lipid control; poor blood pressure & lipid control; and poor glucose, blood pressure & lipid control) with diabetic retinopathy/diabetic macular edema was examined using multiple logistic regression models.

**Results:**

Patients’ mean±standard deviation age was 64.9±11.6 years (57% male). In adjusted models, compared to those with good control of all indicators (n = 99, 18.3%), the odds ratio (95% Confidence Interval) of having any diabetic retinopathy was 2.44 (1.34–4.46), 3.75 (1.75–8.07), 4.64 (2.13–10.12) and 2.28 (1.01–5.16) for poor glucose control only; poor glucose & blood pressure control; poor glucose & lipid control; and poor glucose, blood pressure & lipid control, respectively. Correspondingly for diabetic macular edema, they were 3.19 (1.55–6.59); 3.60 (1.58–8.22); 2.76 (1.18–6.44); and 3.01 (1.18–7.67), respectively. Odds were not significantly increased for other indicators.

**Discussion:**

Compared to individual indicators of poor diabetes control, risk of diabetic retinopathy and diabetic macular edema increased three to fourfold with a combination of these indicators. Targeting combined diabetes control indicators is important to reduce risk of diabetic retinopathy/diabetic macular edema.

## Introduction

Diabetic retinopathy (DR) is a common microvascular complication of diabetes that affects about a third of all people with diabetes[1] and, together with diabetic macular edema (DME), which can occur at any stage of DR,[2] is accountable for 4.8% of the 37 million cases of blindness worldwide.[3] DR can significantly affect quality of life, particularly at the vision-threatening stages (i.e. severe non-proliferative DR, proliferative DR, and clinically significant DME),[4] with substantial public health implications in terms of resources allocated to screening and management.[5]

The association between suboptimal glycemic control and the development and progression of DR and DME is well established.[6] Similarly, strong evidence suggests that hypertension[7] and dyslipidaemia[8] are important risk factors for severity of DR and DME, although these relationships are less well established than for hyperglycemia.[9–12] Based on these epidemiological and clinical trial data, current international (e.g., International Council of Ophthalmology) and national (e.g., Australian National Health and Medical Research Council) guidelines suggest systemic control of these risk factors for the prevention and management of DR in all people with diabetes.[13, 14]

While the importance of achieving optimal glycemic, blood pressure (BP), and serum lipids control has been emphasized, the relative importance and incremental risk of having one, two, or all three indicators of poor control on the severity of DR/DME is unclear. For example, it would be useful to understand what proportion of people with diabetes with poor glycemic control also has suboptimal BP and/or lipid control. It would be also useful to understand how much additional risk is associated with having poor BP or poor lipid control in these patients with poor glycemic control. Such analysis is unavailable from most current studies examining risk factors for DR. Therefore, it is difficult to determine if multiple indicators of poor diabetes control are associated with greater risks of DR/DME compared to one indicator only.

Therefore, we explored the incremental odds of having DR and DME in individuals with a combination of poor diabetes control indicators in adults with type 2 diabetes. We hypothesize that compared to individual indicators of poor diabetes control, combined indicators are associated with higher odds of DR and DME. Moreover, as the number of indicators of poor control (e.g. none, one, two or three) increases, the likelihood of having DR and DME also multiplies.

## Materials and methods

### Study design and participants

We conducted a cross-sectional clinical study, the Australian Diabetes Management Project (DMP), which investigated the clinical, behavioural, and psychosocial factors associated with optimal diabetes control in people with and without DR attending a tertiary eye care facility.[15] English speaking adults aged ≥18 years, with type 1 or type 2 diabetes, free of significant hearing and cognitive impairment, and living independently met the DMP inclusion criterion. The 6-item cognitive impairment test[16] assessed patients’ cognitive capacity, and those who failed were excluded from the main data analysis. In this study, we focus on only those with type 2 diabetes as too few people had type 1diabetes to adequately explore our research question. All study procedures adhered to the tenets of the Declaration of Helsinki and written informed consent was obtained from each participant prior to the study assessment. Ethical approval for the study was provided by the Royal Victorian Eye and Ear Hospital Human Research and Ethics Committee (08/815H).

### Testing Protocol

All examinations were conducted at the Centre for Eye Research Australia (CERA) Melbourne, Australia from March 2009 to December 2010 by a trained interviewer. Participants answered a socio-demographic and medical history questionnaire in order to collect data on age, gender, medical history, height, weight, health and lifestyle factors and duration of diabetes.

### Blood collection

A total fasting blood sample of 34.5ml was collected to assess glycosylated haemoglobin (Hb_A1c_) levels, fasting glucose and lipids (total cholesterol [TC], triglyceride [TG], low density lipoprotein (LDL) and high density lipoprotein (HDL). All biochemical parameters were analysed at Melbourne Pathology, Melbourne, Australia.

### Blood pressure (BP) measurements

A BP assessment was completed on each individual using an automated BP machine, model 5200-103Z (Welch Allyn, New Zealand). The average of two separate measurements was recorded for systolic (SBP), diastolic (DBP) and heart rate. In cases where there was a difference of 10mmHg for SBP or 5mmHg for DBP or greater, a third measurement was taken. The closest two BP measurements were then averaged.

### Individual and combined indicators of diabetes control

Three individual indicators of good diabetes control were used that were defined according to standard established definitions from international guidelines and literature[17–19] namely (1) glycemic control (Hb_A1c_<7%); (2) BP control (SBP/DBP<130/80mmHg); (3) lipid control (TC:HDL<4.0). From these, three individual indicators of poor control were defined: glucose control (Hb_A1c_≥7%); BP control (SBP/DBP≥130/80mmHg); and lipid control (TC:HDL≥4.0). Four combined indicators of poor diabetes control were also examined, namely (1) glycemic & BP control; (2) glycemic & lipid control; (3) BP & lipid control; and (4) glycemic, BP, & lipid control. Current medications were collected for each participant and then subsequently classified as anti-diabetic, anti-hypertensive, and lipid lowering medication for data analysis.

### DR and DME assessment

Two-field (macula and optic disc) dilated fundus photos were captured using a non-mydriatic retinal camera (Cannon CR6-45NM), Cannon Inc, Japan. DR grading was based on the Early Treatment Diabetic Retinopathy Study (ETDRS) and Multi-Ethnic Study of Atherosclerosis (MESA) Digital Grading Protocol.[15] DR severity was classified into: 1) non-proliferative DR (NPDR) and (2) proliferative DR (PDR). NPDR was further grouped into mild, moderate and severe, each stage having defined retinal pathologic signs. For the purpose of our analysis, DR severity was categorized into mild/moderate NPDR and severe NPDR/PDR.

DME was determined using fast macular scans (right and left eye) with retinal map analysis (fast macular thickness map, retinal thickness/Vol Tabular), as well as retinal nerve fibre layer scans (RNFL 3.4mm/average RNFL thickness), (Stratus Model 3000, Carl Zeiss Meditec, Inc., North Ryde, NSW, Australia).

### Statistical analysis

Participants’ demographics and baseline characteristics were summarised by the mean and standard deviation (SD) for normally distributed continuous data, or median and inter-quartile range for skewed data, and counts and percentages for categorical data. Key covariables included age, gender, body mass index (BMI) (kg/m^2^), annual income (<AUD$30,000/≥AUD$30,000), education level (<14 years/≥14 years), smoking status (non-smoker/current or past smoker), duration of diabetes, insulin use (yes/no), presence of another diabetes complication (renal, peripheral vascular disease, neuropathy), presence of co-morbidity (angina, arrhythmia, stroke, asthma, anaemia, migraine, arthritis, osteoporosis), lipid profile (TG, LDL), fasting glucose, and microalbuminuria.

Diabetes control outcomes and demographic and clinical variables were compared in those with and without DR/DME. Poor diabetes control in participants with and without DR/DME was compared using the Chi-square test of association. Multiple logistic and multinomial logistic regression models were used to explore the association between individual and combined indicators of poor diabetes control and presence of DR and DME, and severity of DR, respectively. Models were adjusted for variables found to be significant in univariate analysis (p<0.1), including age, gender, duration of diabetes, HDL, presence of comorbidities, and presence of other diabetes complications. Chi-square was used to test if the differences in odds ratios (ORs) for each of the significant diabetes control variables were significant (p<0.05). A likelihood ratio test was conducted to determine if there was a linear trend for the differences in ORs for each diabetes control variable (p>0.05 if there is a linear trend present). To determine if the associations were confounded by medication use, we conducted the same analyses adjusting for anti-diabetic, anti-hypertensive and lipid lowering medication in those with medication data available (n = 424; 69.2%).

As regression models alone do not provide information on the relative importance of predictors especially when they are correlated, we conducted dominance analysis.[20] By accounting for a variable’s direct, total and partial effects in terms of its contribution to overall variance and model fit statistics, dominance analysis can determine the relative importance of the independent variables in the logistic model. Dominance analysis is useful in health care decision making as it provides a ranking of predictors from ‘most important’ to ‘least important’.[21]

All probabilities quoted are two-sided and all statistical analyses were undertaken using Stata version 14.1 (StataCorp, College Station, TX).

## Results

**Fig 1:**
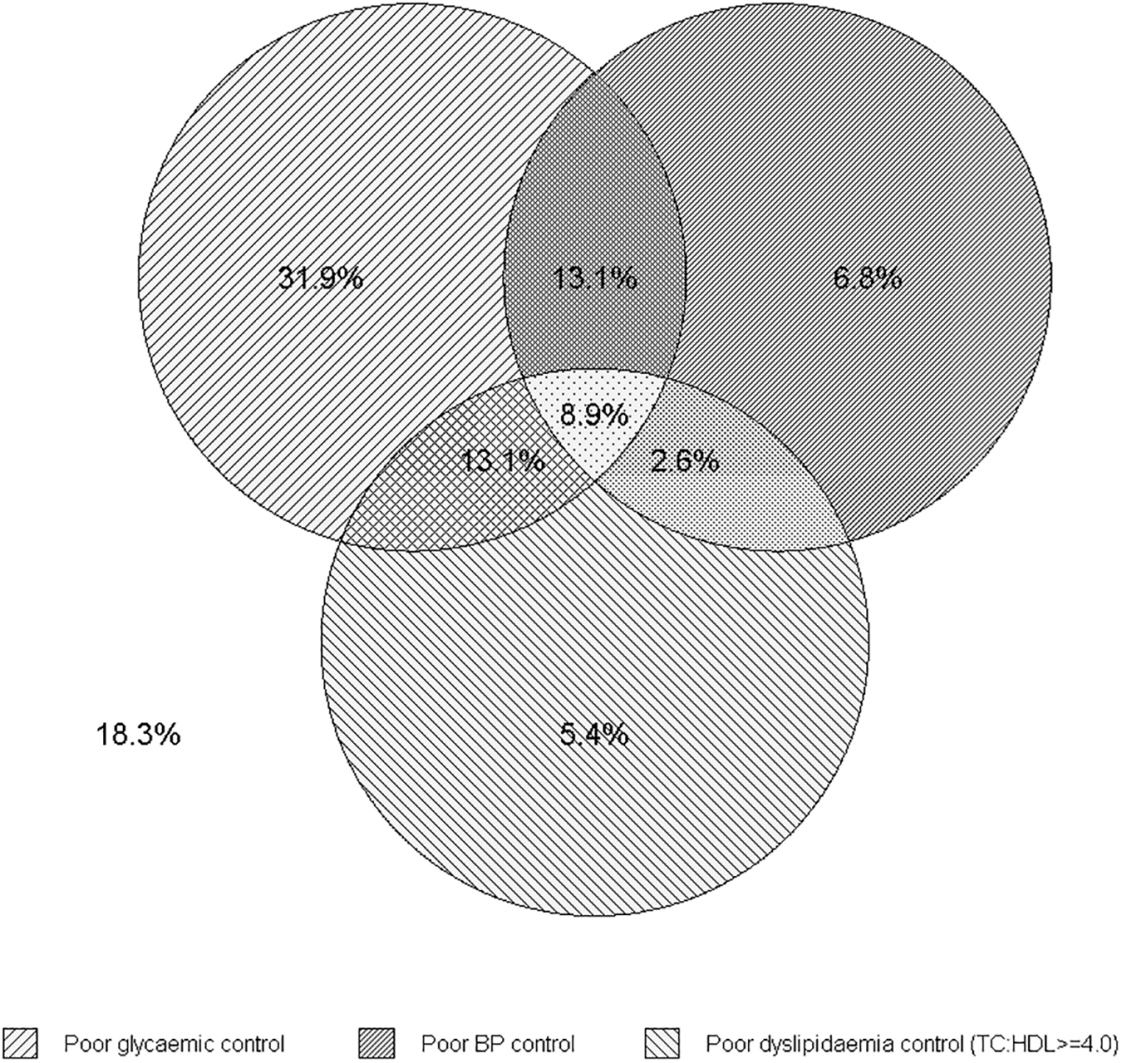
Proportion of participants with individual and combined indicators of poor diabetes control. This figure shows that 8.9% had poor glycemic, poor BP and poor lipid control, while 18.3% had good control of all three indicators. Note: 71 participants had missing data for either glycemic, BP or lipid control and were not included in this figure (total n = 542).

Of the 613 patients (mean±SD age 66.0±10.5 [range 26–89] years and 57% male), 372 (60.1%) had any DR and 181 (30.5%) any DME. Of those with DR, 213 (57.3%) and 159 (42.7%) had mild/moderate NPDR and severe NPDR/PDR, respectively. Compared to patients without DR, those with the condition were more likely to be male, younger, have longer duration of diabetes, use insulin, have at least one other diabetes complication, and at least one comorbidity (all p<0.05, **Table 1**). Those with DR (**Table 1**) and DME (**S1 Table**) were more likely to have poor glucose control, poor BP control and poor lipid control, individually as well as in combination (all p<0.05) compared to those without these conditions. A total of 542 (88.4%) patients had complete data for all three indicators of diabetes control. Of these, less than one fifth (n = 99; 18.3) had good control of all three indicators (Hb_A1c_<7%, SBP/DBP<130/80 and TC:HDL<4.0, **Fig 1**).

**Table 1:** Participants’ socio-demographic and clinical characteristics, stratified for no DR and DR*.

Characteristic	No DR (N = 241)†		DR (N = 372)		p-value
	n	%	n	%	
*Categorical variables*					
Gender (male)	137	56.8	275	73.9	<0.001
Insulin use (yes)	37	15.4	179	48.1	<0.001
Anti-diabetic medication use (yes)‡	120	68.6	190	73.4	0.279
Anti-hypertensive medication use (yes)^‡^	79	45.1	84	32.4	0.007
Lipid lowering medication use (yes)^‡^	52	29.7	71	27.4	0.602
At least one comorbidity§	214	88.8	307	82.5	0.034
At least one diabetes complicationǁ	47	19.5	135	36.3	<0.001
DR severity					
Mild/moderate NPDR	-	-	213	57.3	
Severe NPDR/PDR	-	-	159	42.7	
DME (yes)			181	49.9	
Diabetes control indicators					
Any poor glucose control (HbA1c≥7%)	110	45.6	275	73.9	<0.001
Any poor BP control (SBP/DBP≥ 130/80mmHg)	73	30.3	121	32.5	0.515
Any poor lipid control (TC:HDL≥4.0)	54	22.4	113	30.4	0.065
Composite diabetes control indicators					
Good glucose, BP & lipid control	61	25.3	38	10.2	
Poor glucose control only	56	23.2	117	31.4	<0.001
Poor BP control only	21	8.7	16	4.3	
Poor lipid control only	13	5.4	16	4.3	
Poor glucose & lipid control	16	6.6	55	14.8	
Poor glucose & BP control	16	6.6	55	14.8	
Poor BP & lipid control	7	2.9	7	1.9	
Poor glucose, BP & lipid control	15	6.2	33	8.9	
Missing	36	14.9	35	9.4	
*Continuous variables*¶,#	Mean /median**	*SD* */IQR*	Mean /median**	*SD* */IQR*	*p-value*
Age, years	68.0	10.6^	64.6	10.3	<0.001
Duration of diabetes, years	8.0	11.0	16.0	12.0	<0.001
HbA1c % (mmol/mol)	7.0 (53)	2.5	7.8 (62)	1.6	<0.001
Fasting plasma glucose, mmol/L	7.0	2.5	8.3	3.8	<0.001
Total cholesterol, mmol/L	4.5	1.5	4.3	1.4	0.033
HDL cholesterol mmol/L	1.3	0.5	1.2	0.5	<0.001

*Data are not shown for income, education level, smoking status, use of hypertensive or lipid medication, BMI, triglycerides, and LDL because values were not significantly different between the two groups.

†Chi-square test was used to assess for difference in frequency distributions between DR and no DR

‡ Denominator is 175 for no DR and 259 for DR (total sample with medication data = 434)

§Includes: hypertension, heart attack/angina, irregular heartbeat, stroke, high cholesterol, asthma, anaemia, migraine, arthritis, osteoporosis

ǁIncludes: nephropathy, peripheral vascular disease, neuropathy

¶Student’s unpaired t-test was used for the comparison of continuous normally distributed variables

#Wilcoxon rank-sum test was used for the comparison of skewed distributed variables

**Characteristics were expressed as the median (interquartile range (IQR)) for non-normally distributed continuous variables

^Age range was 29–88 and 26–89 years for those without and with DR, respectively.

BP = Blood pressure; BMI = body mass index; DBP = Diastolic blood pressure; DR = Diabetic retinopathy; DME = Diabetic macular oedema; HDL = High density lipoprotein; IQR = Interquartile range; LDL = Low density lipoprotein; NPDR = Non proliferative diabetic retinopathy; PDR = Proliferative diabetic retinopathy; SD = Standard Deviation; TC = Total cholesterol.

For the individual indicators of control, 31.9%, 6.8% and 5.4% had poor glucose control, poor BP control and poor lipid control, respectively. Regarding the combined indicators of control, 13.1%, 13.1%, 2.6%, and 8.9% had poor glucose & BP control, poor glucose & lipid control, poor BP & lipid control, and poor glucose, BP & lipid control, respectively (**Fig 1**). In those with available medication data, 207 (73.4%) had poor glucose control and were taking anti-diabetic medications; 54 (40.9%) had poor BP control and were taking anti-hypertensive medication; and 26 (20.8%) had poor lipid control and were taking lipid lowering medication (**S2 Table**).

In adjusted models, compared to those with three good control indicators, the odds (OR) of having any DR (95% CI) was 2.44 (1.34–4.46), 3.75 (1.75–8.07), 4.64 (2.13–10.12) and 2.28 (1.01–5.16) for poor glucose control only, poor glucose & lipid control, poor glucose & BP control; and poor glucose, BP & lipid control, respectively (all p<0.05, **Table 2, S1 Fig**). The difference between these ORs was also significant; for example the p-values for the difference between the OR for poor glucose control only (2.44) and poor glucose & lipid control (3.75), and between poor glucose & lipid control (3.75) and poor glucose & BP control (4.64) were p = 0.001 and p<0.001, respectively. A very similar pattern was observed when we adjusted for medication use in the sub-sample of participants with medication data (**S3 Table**). Overall, these data suggest there is a significant increase in odds of DR for a patient with poor lipid or poor BP control *in addition to* poor glucose control, compared to just glucose control alone. In contrast, poor BP control alone, poor lipid control alone, and poor BP & lipid control combined were not independently associated with increased odds of DR.

**Table 2:** Association between individual and combined indicators of diabetes control and presence of DR and presence of DME.

	DR				DME			
*Diabetes control indicators*	*Unadjusted OR*	*p-value*	*Adjusted OR* ^*^	*p-value*	*Unadjusted OR*	*p-value*	*Adjusted OR* ^*^	*p-value*
Good glucose, BP & lipid control†	1.00 (reference)	-	1.00 (reference)	-	1.00 (reference)		1.00 (reference)	
Poor glucose control only	**3.35 (2.00, 5.61)**	**<0.001**	**2.44 (1.34, 4.46)**	**0.004**	**4.11 (2.08, 8.11)**	**<0.001**	**3.19 (1.55, 6.59)**	**0.004**
Poor BP control only	1.22 (0.57, 2.63)	0.607	1.79 (0.82, 3.92)	0.145	1.35 (0.47, 3.92)	0.576	1.60 (0.51, 4.99)	0.145
Poor lipid control only	1.98 (0.86, 4.56)	0.111	1.65 (0.56, 4.89)	0.366	2.33 (0.82, 6.65)	0.113	2.05 (0.62, 6.79)	0.237
Poor glucose & lipid control	**5.52 (2.77, 10.99)**	**<0.001**	**3.75 (1.75, 8.07)‡**	**0.001**	**5.29 (2.47, 11.37)**	**<0.001**	**3.60 (1.58, 8.22)** ¶	**0.002**
Poor glucose & BP control	**5.52 (2.77, 10.99)**	**<0.001**	**4.64 (2.13, 10.12)§**	**<0.001**	**3.57 (1.63, 7.86)**	**<0.001**	**2.76 (1.18, 6.44)** #	**0.019**
Poor BP & lipid control	1.61 (0.52, 4.94)	0.409	1.55 (0.48, 5.02)	0.463	2.80 (0.76, 10.37)	0.123	2.34 (0.64, 8.60)	0.200
Poor glucose, BP & lipid control	**3.53 (1.69, 7.35)**	**0.001**	**2.28 (1.01, 5.16)ǁ**	**0.047**	**4.75 (2.05, 11.01)**	**0.001**	**3.01 (1.18, 7.67)** **	**0.021**

Bolded values indicate significant results.

*Adjusted for age, gender, duration of diabetes, high density lipoprotein, presence of comorbidities, and presence of other diabetes complications.

† Likelihood-ratio test for linear trend tests of ORs for individual and combined indicators of diabetes control: p = 0.024 (DR model) and p = 0.05 (DME model)

‡ Significantly greater than poor glucose control only: p = 0.001

§ Significantly greater than poor glucose & lipid control: p<0.001

ǁ Significantly greater than poor glucose control only: p = 0.008

¶ Significantly greater than poor glucose control only: p = 0.004; # Significantly smaller than poor glucose control only: p<0.001

** Significantly smaller than poor glucose control only: p = 0.007

BP = Blood pressure; DR = Diabetic retinopathy; DME = Diabetic macular edema; OR = Odds ratio

Poor glucose & lipid control, and poor glucose & BP control were also associated with increased odds of mild/moderate NPDR and severe NPDR/PDR compared to no DR in adjusted models. However, unlike any DR, combined poor glucose, BP & lipid control was not independently associated with DR severity (**S4 Table**).

Contrary to our second hypothesis, the likelihood ratio test indicated that the trend for the diabetes control ORs was non-linear (p = 0.024), suggesting that the odds of having DR did not linearly increase as the number of poor diabetes control indicators increased.

In adjusted models looking at the association between diabetes control indicators and DME (**Table 2**), the odds of having DME were 3.19 (1.55–6.59) for poor glucose control only; 3.60 (1.58–8.22) for poor glucose & lipid control; 2.76 (1.18–6.44) for poor glucose & BP control; and 3.01 (1.18–7.67) for poor glucose, BP & lipid control, respectively, compared to those with good control of all three indicators (**S2 Fig**). The differences in ORs compared to glucose control only were all significant (p<0.05), and there was a borderline linear trend between the ORs (likelihood ratio test p = 0.05). The ORs increased in magnitude when we adjusted for medication use (**S3 Table**).

**Table 3** shows the results of the dominance analysis. For DR, duration of diabetes and age were the top two ranking variables among the 13 variables evaluated and accounted for ~60% of the predicted variance (51% and 10%, respectively). Of the seven diabetes control indicators, poor glucose & BP control, poor glucose & lipid control, poor glucose control only, and poor glucose, BP & lipid control ranked third, fourth, sixth and tenth, respectively, which supports the magnitude of the ORs observed from the multiple regression analyses (**Table 2**). The remaining four diabetes control indicators were only ranked 9, 10, 12 and 13 (**Table 3**). For DME, age (29%) was ranked first and diabetes duration (28%) second, while poor glucose control only, poor glucose & lipid control, poor glucose, BP & lipid control and poor glucose & BP control were ranked 4, 5, 6 and 7, respectively, again reflecting the magnitude of the ORs resulting from the multiple regression analyses (**Table 2**).

**Table 3:** Importance of associated factors for DR and DME*.

DR		DME		
Variables	Standardized weight†	Rank	Standardized weight†	Rank
Diabetes duration	0.5061	1	0.2764	2
Age	0.1020	2	0.2932	1
Poor glucose & BP control	0.0860	3	0.0263	7
Poor glucose & lipid control	0.0719	4	0.0834	5
Gender	0.0617	5	0.0142	9
Poor glucose control only	0.0465	6	0.0861	4
At least one diabetes complication‡	0.0462	7	n/a	n/a
High density lipoprotein	0.0258	8	n/a	n/a
Poor BP control only	0.0175	9	0.0249	8
Poor glucose, BP & lipid control	0.0166	10	0.0397	6
At least one comorbidity§	0.0117	11	0.1458	3
Poor BP & lipid control	0.0042	12	0.0045	11
Poor lipid control only	0.0037	13	0.0055	10

*Ranking ordered by DR

†Standardized weight is the general dominance weight from McFadden R^2^ normed or standardized to be out of 100%. The standard weights might not add up to 1 due to rounding errors.

‡ Includes: hypertension, heart attack/angina, irregular heartbeat, stroke, high cholesterol, asthma, anaemia, migraine, arthritis, osteoporosis

§ Includes: nephropathy, peripheral vascular disease, neuropathy

BP = Blood pressure; DME = Diabetic macular edema; DR = Diabetic retinopathy

## Discussion

Our study found that less than one in five persons attending a tertiary eye care facility achieved the combination of optimal glucose control, BP control and lipid control. In contrast, nearly one in ten participants had poor control of all three indicators of diabetes control, and nearly two thirds had poor glucose control, suggesting that diabetes control in adults with type 2 diabetes in Australia remains extremely poor, consistent with other studies.[22, 23] Compared to those with good control of all three indicators, persons with suboptimal BP control *and* glucose control; and those with suboptimal lipid control *and* glucose control were nearly four to five times more likely to have DR, respectively, while those with poor glucose control only were 2.5 times more likely. Similarly, the odds of having DME were significantly higher in those with poor glucose control *and* poor lipid control compared to those with poor glucose control alone. Our study suggests that while poor BP control or poor lipid control on their own do not greatly increase risk of DR, they amplify the risk when combined with poor glucose control. These findings support the current guidelines that suggest a multifactorial systemic management plan for people with diabetes is needed to manage DR and DME.

The proportion of participants achieving all three indicators of good control was only 18.3% in our study, which is higher than found in previous studies in Australia and elsewhere, which have reported 13.0%[24, 25] and 13.6%.[17] The inter-study discrepancy could be explained by differences in study design (e.g. population-based vs. clinical), and study population (Indian[17] and Israeli[24] participants). Similarly, definitions of the indicators of diabetes control varied, particularly for poor lipid control, which was defined as TC:HDL≥4.0 in our study, and LDL≥100mg/dl[17, 24] and TC≥5.5 mmol/l[25] in the others. In the present study, optimal glucose control was achieved by only 37% of participants, which is similar to other studies,[17, 22, 26, 27] although lower than the 1999–2000 AusDiab population based study where 57% achieved the appropriate glycemic target.[25] This may be due to the population-based study design of AusDiab compared to our clinic based study.

Of the individual indicators of poor control in our study, only poor glucose control was independently associated with higher odds of DR and DME. This differs from other studies which have found that hypertension[7, 28] and hyperlipidemia[8] are associated with the presence or progression of DR and DME; however, these studies did not assess persons with *only* poor BP control or poor lipid control and it is extremely likely that some participants also had poor glucose control, suggesting that poor BP control and poor lipid control are only independently associated with DR/DME in combination with poor glucose control. When combined with poor glucose control, poor lipid control in our study was associated with an incrementally and significantly higher risk of DR and DME than all three individual control indicators. Interestingly, when poor BP control was combined with poor glucose control, it resulted in higher risk of DR but lower risk of DME, compared to poor glucose control only. This could be because 20% of those who had good lipid control in our sample were taking lipid-lowering medication which may be protective for DME.[29]

The implications of these results for clinicians and researchers are twofold. First, of the three diabetes control indicators, poor glucose control remains the most important risk factor for both DR and DME. Second, while poor BP control and poor lipid control individually are not independent risk factors for DR/DME, when combined with poor glucose control they compound the risk of DR. Similarly, when in combination with poor glucose control, poor lipid control amplifies the risk of DME. However, given that the influence of multiple risk factors for DR may depend on the severity of the disease,[30] our results must be interpreted with caution.

Few studies have specifically investigated the risk of DR/DME in people with diabetes using individual *and* combined indicators of diabetes control.[17, 31] However, our findings support previous studies which have found that combined reduction of diabetes control indicators reduces the risk of cardiovascular and microvascular complications.[32] For example, the Action in Diabetes and Vascular Disease (ADVANCE) study found that participants within a combination treatment group of intensive BP and glycemic control had significantly reduced risk of cardiovascular disease and renal events compared to those in the intensive glycemic control group alone.[33] Similarly, the Treating to New Targets (TNT) trial found that the benefits of statin treatment were substantially higher in patients with better glycemic control.[34] Moreover, Gaede and colleagues reported that multifactorial intervention (i.e. tight glycemic control, and use of hypertensive and lipid lowering medications) resulted in significant reductions in CVD and microvascular complications, including DR, compared with usual care.[35]

Contrary to our hypothesis, although having poor control of all three indicators was an independent risk factor for DR and DME, the OR was lower than the ORs for poor glucose control alone and other combined indicators. Moreover, the likelihood ratio test suggested that the relationship between the number of indicators of poor control and DR/DME risk was not linear. This unexpected pattern persisted even when adjusting for medication use in a sub-sample of participants, suggesting that the observed associations are not dependent on treatment status. Similarly, although combined poor glucose, BP & lipid control was associated with increased risk of non-VTDR and VTDR in univariate analysis, the association was no longer significant in adjusted models. Although counterintuitive, this finding is unlikely to be spurious as we replicated the pattern in a population-based sample of 2208 Singaporean Malays, Indians and Chinese with diabetes from the Singapore Epidemiology of Eye Diseases (SEED) study[36] (data presented at the Association for Research in Vision and Ophthalmology [ARVO] 2015 conference in Colorado, USA). For example, in adjusted models, compared to those with three good control indicators, the OR of having any DR (95% CI) was 1.56 (1.07–2.25), 1.92 (1.37–2.68), 1.85 (1.18–2.90) and 1.69 (1.18–2.43) for poor glucose control only, poor glucose & lipid control, poor glucose & BP control; and poor glucose, BP & lipid control, respectively (all p<0.05).[37] This somewhat counterintuitive finding could be because those with poor control of the three indicators were more likely subjected to more frequent and earlier screening and prompt referral meaning that DR was more likely to be detected early. It may also be related to the relatively small proportion of participants with all three indicators of poor control 9% (n = 55) which may have reduced our ability to detect an association, especially in our analysis of DR severity. More research in a larger sample size with more participants in this three indicator group is needed to further explore the effect.

The derived standardised weight estimates produced from our dominance analysis demonstrated that 60% of the predicted variance in DR/DME risk was attributable to two top-ranked predictors, namely longer duration of diabetes and older age, while all seven individual and combined indicators of diabetes control contributed only 25%. This finding suggests that much of the risk associated with DR/DME stems from non-modifiable risk factors and highlights the importance of preventing diabetes for as long as possible to reduce duration. Of the modifiable risk factors, combined poor glucose control & BP control, and combined poor glucose control & lipid control were the highest ranking for DR, together contributing about 16% of the variance and suggesting that focussing on reducing BP and lipids as well as blood glucose is important. Importantly, 73%, 41% and 21% of people with poor glucose control, poor BP control and poor lipid control in our study were currently taking medication for these conditions. Ophthalmologists should be aware that even if patients are being treated, they may still have poor control of these risk factors and may require additional follow-up or referral. For DME, poor glucose control only and combined poor glucose control & lipid control were the highest ranking variables (17% variance) highlighting the importance of lipid control in combination with glucose control for DME.

Strengths of our study include a well characterised sample with differing levels of DR, a comprehensive collection of medical and sociodemographic parameters, objective assessments of DR and novel statistical analyses. Limitations include potential selection biases from our focused recruitment from specialized retinal clinics meaning that our results may not be generalizable to the broader population with diabetes. Furthermore, owing to small sample sizes we may have had reduced power to determine the relationship between diabetes control indicators and DR severity. Our current findings are based on cross-sectional data which means that establishing causal relationships is not possible and we cannot determine how sustained poor control impacts disease risk or progression. Finally, as international diabetes control guidelines now include an individualized target of metabolic control dependent on age and life expectancy, our findings may not apply to all people with diabetes.

In summary, we found high rates of poor systemic diabetes control in our clinical sample of individuals with type 2 diabetes. Compared to good diabetes control, combined indicators of poor glucose control, and poor BP control or poor lipid control were associated with an incrementally and significantly higher risk of both presence and severity of DR than poor glucose control alone, and combined poor glucose and lipid control was associated with significantly higher risk of DME. While glucose control remains the cornerstone of optimal diabetes management, BP control and lipid control are also important in persons with poor glucose control. This is often missed in clinical settings. Our findings reinforce this message to patients to prevent vision loss from DR and DME.

## Supporting information

S1 Fig Association between individual and combined indicators of diabetes control and risk of diabetic retinopathy in multiple logistic regression models. BPC = Blood pressure control; BP&LC = Blood pressure and lipid control; GC = Glucose control; G&BPC = Glucose and blood pressure control; G&BP&LC = Glucose, blood pressure and lipid control; G&LC = Glucose and lipid control; LC = Lipid control.
(TIFF)

S2 Fig Association between individual and combined indicators of diabetes control and risk of diabetic macular edema in multiple logistic regression models. BPC = Blood pressure control; BP&LC = Blood pressure and lipid control; GC = Glucose control; G&BPC = Glucose and blood pressure control; G&BP&LC = Glucose, blood pressure and lipid control; G&LC = Glucose and lipid control; LC = Lipid control.
(TIFF)

S1 Table Distribution of diabetes control indicators by DME status in patients with type 2 diabetes (n = 602). (DOCX)

S2 Table Medication use among those with good and poor glycemic, blood pressure and lipid control. (DOCX)

S3 Table Association between individual and combined indicators of diabetes control, and DR (n = 381)* and DME (n = 377)†, adjusted for medication use in a subsample of participants‡. (DOCX)

S4 Table Association between individual and combined indicators of diabetes control and severity of diabetic retinopathy. (DOCX)
