# Supplementary figures and images for: Combined poor diabetes control indicators are associated with higher risks of diabetic retinopathy and macular edema than poor glycemic control alone
Source: PLoS One. 2017 Jun 29;12(6):e0180252. doi: 10.1371/journal.pone.0180252 (PMC5491170; doi:10.1371/journal.pone.0180252)

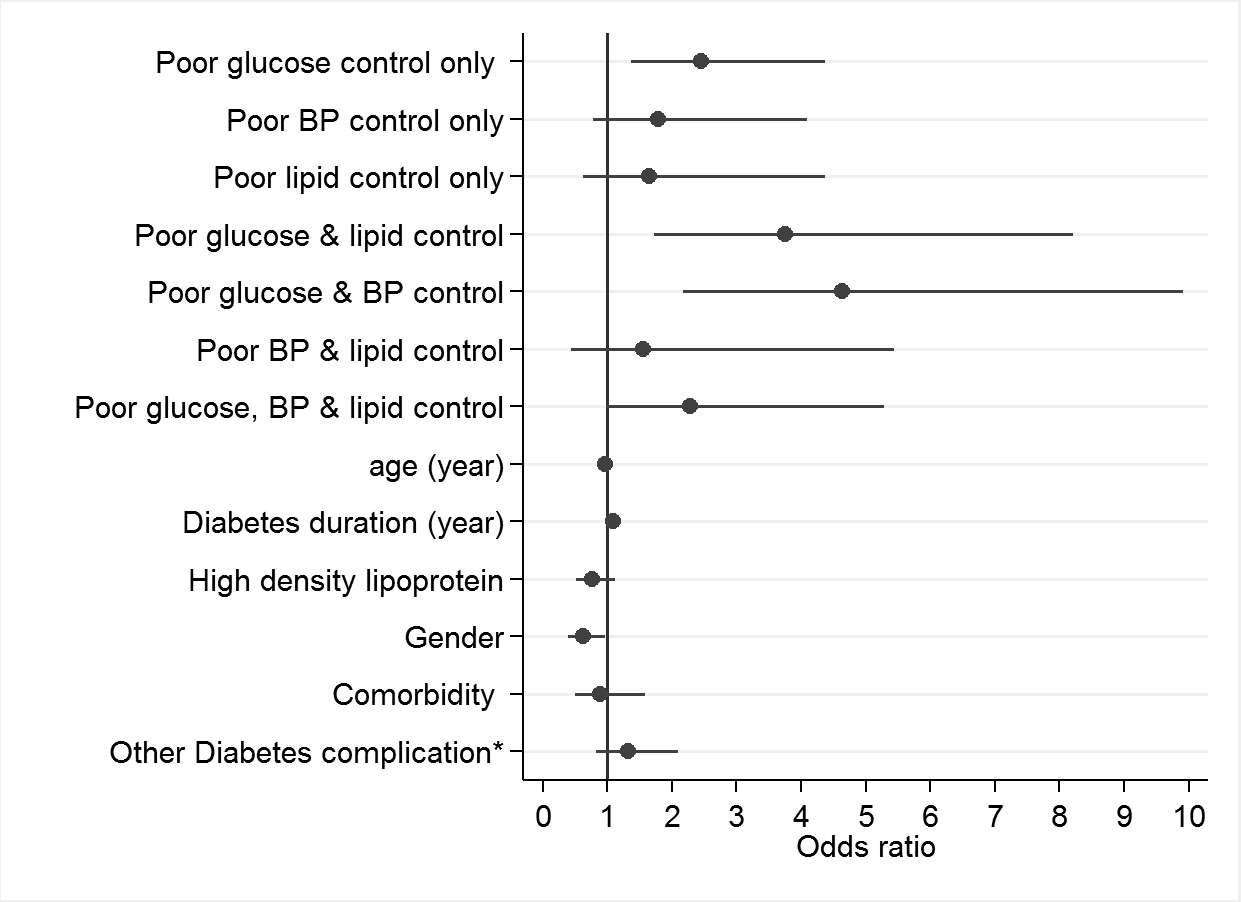

Supplement: S1 Fig — BPC = Blood pressure control; BP&LC = Blood pressure and lipid control; GC = Glucose control; G&BPC = Glucose and blood pressure control; G&BP&LC = Glucose, blood pressure and lipid control; G&LC = Glucose and lipid control; LC = Lipid control. (TIFF) [file pone.0180252.s001.tiff]

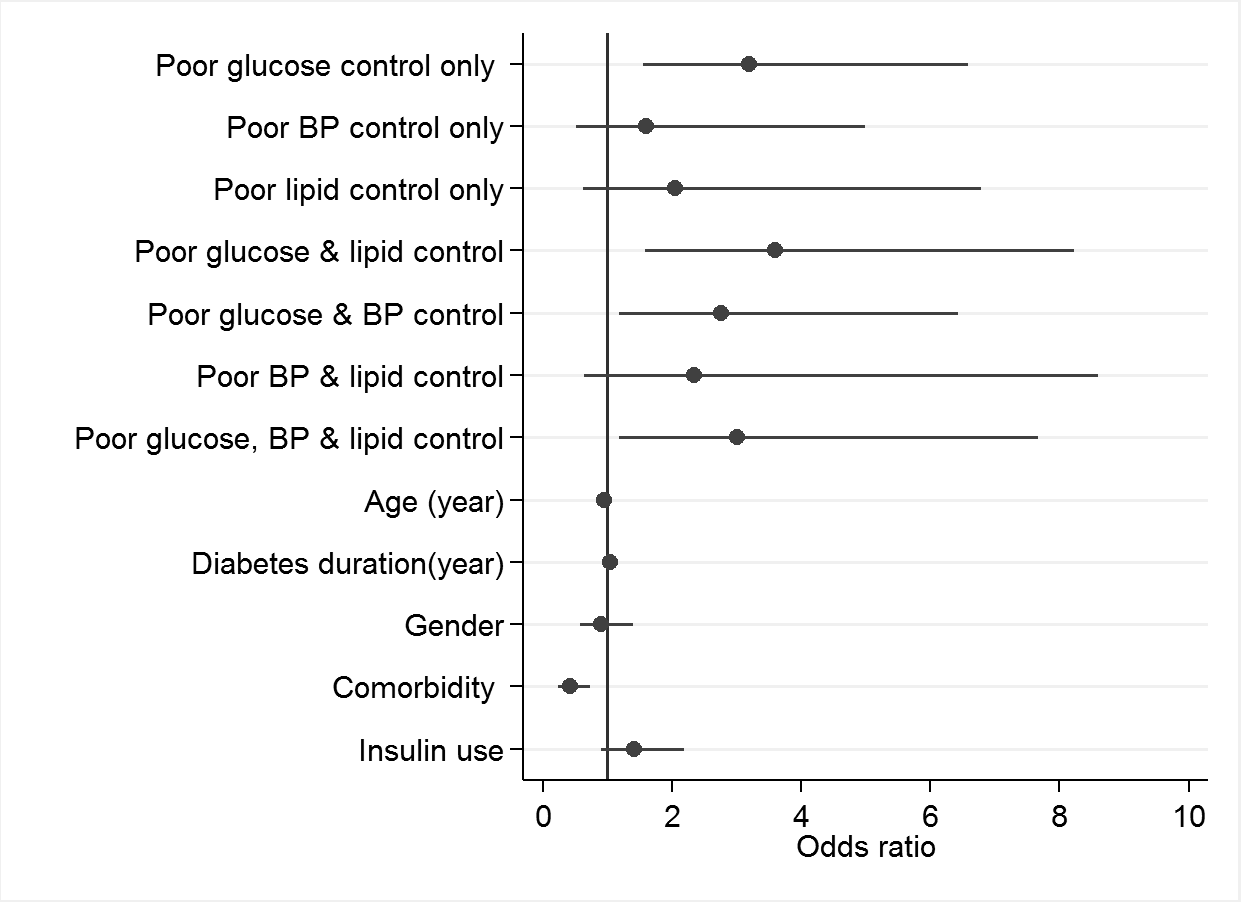

Supplement: S2 Fig — BPC = Blood pressure control; BP&LC = Blood pressure and lipid control; GC = Glucose control; G&BPC = Glucose and blood pressure control; G&BP&LC = Glucose, blood pressure and lipid control; G&LC = Glucose and lipid control; LC = Lipid control. (TIFF) [file pone.0180252.s002.tiff]
