# Supplementary material for: Combined poor diabetes control indicators are associated with higher risks of diabetic retinopathy and macular edema than poor glycemic control alone
Source: PLoS One. 2017 Jun 29;12(6):e0180252. doi: 10.1371/journal.pone.0180252 (PMC5491170; doi:10.1371/journal.pone.0180252)
Supplement: S1 Table — (DOCX) [file pone.0180252.s003.docx]

| **S1 Table.** **Distribution of diabetes control indicators by DME status in patients with type 2 diabetes (n=602)** | | | | | |
| --- | --- | --- | --- | --- | --- |
| **Diabetes control indicators** | **No DME (n=421)** | | **DME (n=181)** | | **p-value** |
|  | **n** | **%** | **n** | **%** |  |
| Any poor glycaemic control (HbA1C≥7%) | 224 | 60.3 | 147 | 83.1 | <0.001 |
| Any poor BP control (SBP/DBP≥ 130/80mmHg) | 134 | 32.5 | 55 | 30.6 | <0.001 |
| Any poor dyslipidaemia control (TC:HDL≥4.0) | 103 | 27.0 | 62 | 36.5 | 0.025 |
| **Composite** **Diabetes control indicators** |  |  |  |  |  |
| Good glucose, BP & lipid control | 84 | 22.8 | 12 | 7.2 | <0.001 |
| Poor glucose control only | 109 | 29.5 | 64 | 38.6 |  |
| Poor BP control only | 31 | 8.4 | 6 | 3.6 |  |
| Poor lipid control only | 21 | 5.7 | 7 | 4.2 |  |
| Poor glucose & lipid control | 41 | 11.1 | 31 | 18.7 |  |
| Poor glucose & BP control | 45 | 12.2 | 23 | 13.9 |  |
| Poor BP & lipid control | 10 | 2.7 | 4 | 2.4 |  |
| Poor glucose, BP & lipid control | 28 | 7.6 | 19 | 11.4 |  |
| BP=Blood pressure; DBP=Diastolic BP; DME=Diabetic macular edema; HDL=High density lipoprotein; SBP=Systolic BP; TC=Total cholesterol | | | | | |
