# Supplementary material for: Combined poor diabetes control indicators are associated with higher risks of diabetic retinopathy and macular edema than poor glycemic control alone
Source: PLoS One. 2017 Jun 29;12(6):e0180252. doi: 10.1371/journal.pone.0180252 (PMC5491170; doi:10.1371/journal.pone.0180252)
Supplement: S2 Table — (DOCX) [file pone.0180252.s004.docx]

| **S2 Table. Medication use among those with good and poor glycemic, blood pressure and lipid control** | | | | | | | | |
| --- | --- | --- | --- | --- | --- | --- | --- | --- |
| *Medication use* | **Poor glucose control*** | **Good glucose control*** |  | **Poor BP control**† | **Good BP control**† | **Poor lipid control**‡ | **Good lipid control**‡ |  |
| Anti-diabetic (yes) | 207 (73.4) | 89 (68.5) |  |  |  |  |  |  |
| Anti-diabetic (no) | 75 (26.6) | 41 (31.5) |  |  |  |  |  |  |
| Anti-hypertensive (yes) |  |  |  | 54 (40.9) | 108 (36.7) |  |  |  |
| Anti-hypertensive (no) |  |  |  | 78 (59.1) | 186 (63.3) |  |  |  |
| Lipid lowering (yes) |  |  |  |  |  | 26 (20.8) | 83 (20.2) |  |
| Lipid lowering (no) |  |  |  |  |  | 99 (79.2) | 328 (79.8) |  |
| *Poor glucose control defined as HbA_1C_≥7%; good glucose control defined as <HbA_1C_7%  †Poor BP control defined as ≥130/80mmHg; good BP control defined as <130/80mmHg  ‡ Poor lipid control defined as TC:HDL≥4.0; good lipid control defined as TC:HDL<4.0 | | | | | | | | |
