# Supplementary material for: Combined poor diabetes control indicators are associated with higher risks of diabetic retinopathy and macular edema than poor glycemic control alone
Source: PLoS One. 2017 Jun 29;12(6):e0180252. doi: 10.1371/journal.pone.0180252 (PMC5491170; doi:10.1371/journal.pone.0180252)
Supplement: S3 Table — (DOCX) [file pone.0180252.s005.docx]

| **S3 Table. Association between individual and combined indicators of diabetes control, and DR (n=381)* and DME (n=377)†, adjusted for medication use in a subsample of participants‡** | | | | |  |
| --- | --- | --- | --- | --- | --- |
| *Diabetes control indicators* | *Adjusted OR* | *p-value* | *Adjusted OR* | *p-value* | |
| Good glucose, BP & lipid control | 1.00 (reference) | - | 1.00 (reference) | - | |
| Poor glucose control only | **2.46 (1.15, 5.21)** | **0.019** | **4.99 (1.99, 12.54)** | **0.001** | |
| Poor BP control only | 1.48 (0.48, 4.51) | 0.494 | 1.40 (0.30, 6.47) | 0.667 | |
| Poor lipid control only | 1.92 (0.60, 6.13) | 0.273 | 3.10 (0.86, 11.16) | 0.083 | |
| Poor glucose & lipid control | **2.93 (1.09, 7.90)**§ | **0.034** | **3.37 (1.20, 9.51)**# | **0.022** | |
| Poor glucose & BP control | **3.79 (1.47, 9.78)**\|\| | **0.006** | **4.07 (1.41, 11.78)**** | **0.010** | |
| Poor BP & lipid control | 1.86 (0.31, 11.28) | 0.501 | 3.14 (0.56, 17.52) | 0.192 | |
| Poor glucose, BP & lipid control | **2.29 (0.78, 6.66)**¶ | **0.129** | **5.03 (1.59, 15.96)**†† | **0.006** | |
| Bolded values indicate significant results.  *Final sample size < 424 due to missing data (n=43) for some variables.  †Final sample size less than 424 due to missing data (n=47) for some variables.  ‡ Adjusted for age, gender, duration of diabetes, high density lipoprotein, presence of comorbidities, presence of other diabetes complications, and use of anti-diabetic, anti-hypertensive, and lipid lowering medication.  §Significantly greater than poor glucose control only: p=0.036; \|\|Significantly greater than poor glucose & lipid control: p=0.014; ¶Significantly greater than poor glucose control only: p=0.06; #Significantly greater than poor glucose control only: p=0.003; **Significantly greater than poor glucose & lipid control: p=0.024; ††Significantly greater than poor glucose control only: p=0.002  BP=Blood pressure; DR=Diabetic retinopathy; DME=Diabetic macular edema; OR=Odds ratio | | | | |  |
