# Supplementary material for: Combined poor diabetes control indicators are associated with higher risks of diabetic retinopathy and macular edema than poor glycemic control alone
Source: PLoS One. 2017 Jun 29;12(6):e0180252. doi: 10.1371/journal.pone.0180252 (PMC5491170; doi:10.1371/journal.pone.0180252)
Supplement: S4 Table — (DOCX) [file pone.0180252.s006.docx]

| **S4 Table. Association between individual and combined indicators of diabetes control and severity of DR** | | | | | |
| --- | --- | --- | --- | --- | --- |
|  | *Diabetes control indicators* | *Unadjusted OR* | *p-value* | *Adjusted OR** | *p-value* |
| No DR (reference) |  | | | | |
| Mild/moderate NPDR | Good glucose, BP & lipid control | 1 |  | 1 |  |
|  | Poor glucose control only | **2.92 (1.62, 5.24)** | **<0.001** | **1.97 (1.03, 3.80)** | **0.041** |
|  | Poor BP control only | 1.05 (0.42, 2.59) | 0.923 | 1.46 (0.59, 3.73) | 0.145 |
|  | Poor lipid control only | 1.88 (0.73, 4.84) | 0.193 | 1.78 (0.57, 5.62) | 0.321 |
|  | Poor glucose & lipid control | **4.27 (1.97, 9.23)** | **<0.001** | **3.73 (1.65, 8.47)** | **0.002** |
|  | Poor glucose & BP control | **5.03 (2.36, 10.74)** | **<0.001** | **4.15 (1.81, 9.52)** | **<0.001** |
|  | Poor BP & lipid control | 1.39 (0.37, 5.19) | 0.620 | 1.54 (0.40, 6.00) | 0.528 |
|  | Poor glucose, BP & lipid control | **2.60 (1.12, 6.06)** | **0.027** | 2.18 (0.84, 5.67) | 0.109 |
| Severe NPDR/PDR | Good glucose, BP & lipid control | 1 |  | 1 |  |
|  | Poor glucose control only | **4.19 (2.06, 5.26)** | **<0.001** | **2.37 (1.07, 5.27)** | **0.034** |
|  | Poor BP control only | 1.56 (0.55, 4.45) | 0.402 | 2.70 (0.92, 7.95) | 0.071 |
|  | Poor lipid control only | 2.17 (0.69, 6.76) | 0.183 | 1.67 (0.42, 6.69) | 0.468 |
|  | Poor glucose & lipid control | **7.92 (3.35, 18.74)** | **<0.001** | **4.53 (1.71, 11.99)** | **0.002** |
|  | Poor glucose & BP control | **6.45 (2.67, 15.56)** | **<0.001** | **4.78 (1.77 (12.99)** | **0.002** |
|  | Poor BP & lipid control | 2.01 (0.46, 8.34) | 0.355 | 1.49 (0.31, 7.28) | 0.619 |
|  | Poor glucose, BP & lipid control | **5.32 (2.12, 13.31)** | **<0.001** | 2.49 (0.84, 7.41) | 0.101 |
| Bolded values indicate significant results.  *Adjusted for age, gender, duration of diabetes, high density lipoprotein, presence of comorbidities, and presence of other diabetes complications.  BP=Blood pressure; DR=Diabetic retinopathy; NPDR=Non-proliferative diabetic retinopathy; OR=Odds ratio; PDR=Proliferative diabetic retinopathy | | | | | |
